# Evaluating the Diagnostic Efficacy of Computed Tomography in Appendicitis Cases With Negative Ultrasound Findings

**DOI:** 10.7759/cureus.69822

**Published:** 2024-09-20

**Authors:** Vishal Vijayakumar, Prabakaran T, Sendhil Sudarsan S, Lokesh Kumar T

**Affiliations:** 1 Department of Radiodiagnosis, Mahatma Gandhi Medical College and Research Institute, Sri Balaji Vidyapeeth University, Pondicherry, IND; 2 Department of General Surgery, Mahatma Gandhi Medical College and Research Institute, Sri Balaji Vidyapeeth University, Pondicherry, IND

**Keywords:** appendicitis, computed tomography, diagnostic accuracy, negative findings, ultrasound

## Abstract

Introduction: Acute appendicitis (AA) is a prevalent cause of abdominal pain, and accurate diagnosis is critical to prevent complications such as perforation. While ultrasound (USG) is often the first imaging modality, its limitations necessitate alternative approaches, particularly in cases where USG results are negative.

Objectives and aims: This study aims to evaluate the diagnostic accuracy of computed tomography (CT) in identifying appendicitis in patients presenting with negative USG findings.

Materials and methods: A prospective observational study was conducted at a tertiary care hospital in Pondicherry, India, involving 70 patients with clinically suspected appendicitis and negative USG results. All patients underwent CT imaging, and findings were analyzed to determine sensitivity, specificity, positive predictive value (PPV), negative predictive value (NPV), and overall accuracy.

Results: Of the 70 patients, 35 were diagnosed with appendicitis based on CT findings, yielding a sensitivity of 100%, a specificity of 65.71%, a PPV of 74.47%, an NPV of 100%, and an overall accuracy of 82.86%. The study also identified other conditions, such as mesenteric lymphadenitis and ureteric calculus, in patients with negative appendicitis diagnoses.

Conclusions: CT is a highly effective imaging modality for diagnosing appendicitis in patients with negative USG results. The use of CT significantly aids in clinical decision-making, reducing the rates of unnecessary surgeries and complications.

## Introduction

The primary purpose of this study is to evaluate the diagnostic efficacy of computed tomography (CT) in identifying acute appendicitis (AA) in patients who present with negative ultrasound (USG) findings. This research aims to determine the sensitivity, specificity, positive predictive value (PPV), negative predictive value (NPV), and overall accuracy of CT in a specific patient population. By analyzing the outcomes of CT imaging in cases where USG results are inconclusive, we aim to establish CT as a reliable alternative diagnostic tool in the clinical evaluation of suspected appendicitis.

Relevance and rationale for the research

AA remains one of the leading causes of abdominal pain requiring surgical intervention [1]. Prompt and accurate diagnosis is essential to prevent complications, significantly affecting patient morbidity and healthcare costs [2,3]. While USG is commonly used as the first-line imaging modality due to its accessibility and safety, its limitations in certain scenarios, such as in patients with atypical presentations or anatomical variations, lead to missed diagnoses and delayed treatment [4]. Misdiagnosis is common in the female population, especially at reproductive age, because of similarities in the clinical presentation of gynecological conditions that mimic AA [5]. Surgeons have accepted a negative appendectomy rate of 20%. This figure remains unchanged today despite the use of modern preoperative diagnostic tests [5]. Similarly, the rate of misdiagnosis for children aged <12 years and <2 years is 57% and 100%, respectively, at first presentation [6].

In clinical practice, negative USG findings can create uncertainty, increasing reliance on surgical exploration, which carries inherent risks. Therefore, exploring the role of CT in these situations becomes crucial. CT has been shown to provide superior anatomical detail and diagnostic accuracy compared to USG, particularly in challenging cases. Given the increasing availability and use of CT in emergency departments, understanding its effectiveness in diagnosing appendicitis when USG results are negative is of paramount importance [7].

This study is particularly relevant as it addresses a critical gap in the existing literature concerning the diagnostic pathway for appendicitis. By investigating the utility of CT in USG-negative patients, we hope to contribute valuable data that can inform clinical guidelines, enhance diagnostic protocols, and ultimately improve patient outcomes in cases of suspected appendicitis.

## Materials and methods

This study employed a prospective observational design conducted over one year, from February 2023 to January 2024, at Mahatma Gandhi Medical College and Research Institute, a tertiary care center in Pondicherry, India, after obtaining ethical approval from the Institutional Human Ethics Committee (approval number: MGMCRI/Res/01/2021/113/IHEC/153). Informed consent was obtained from all participants before their inclusion in the study. The research followed standard protocols for patient management and imaging, ensuring adherence to ethical guidelines throughout the study period.

Inclusion criteria

Included were adults and children aged 0-80 years presenting with clinical signs and symptoms that are indicative of AA, such as right iliac fossa pain, vomiting, and fever, who underwent an abdominal USG that yielded negative findings for appendicitis.

Exclusion criteria

Patients with typical USG findings of AA and medical conditions contraindicating the use of contrast agents (e.g., renal failure or hypersensitivity to contrast media) and pregnant individuals, due to the potential risks associated with imaging, were excluded from the study.

The department is equipped with advanced imaging technology, including a 128-slice CT scanner, allowing for comprehensive imaging evaluations. Patients were referred for imaging from the emergency department and the surgical department, ensuring a diverse cohort representative of the local population. 

The study aimed to gather data on the clinical presentation, imaging findings, and final diagnoses to assess the effectiveness of CT in diagnosing appendicitis in patients with negative USG results. The study utilized a two-step imaging process to diagnose AA in patients, beginning with abdominal USG followed by CT if USG results were negative.

USG protocol

**Figure 1:**
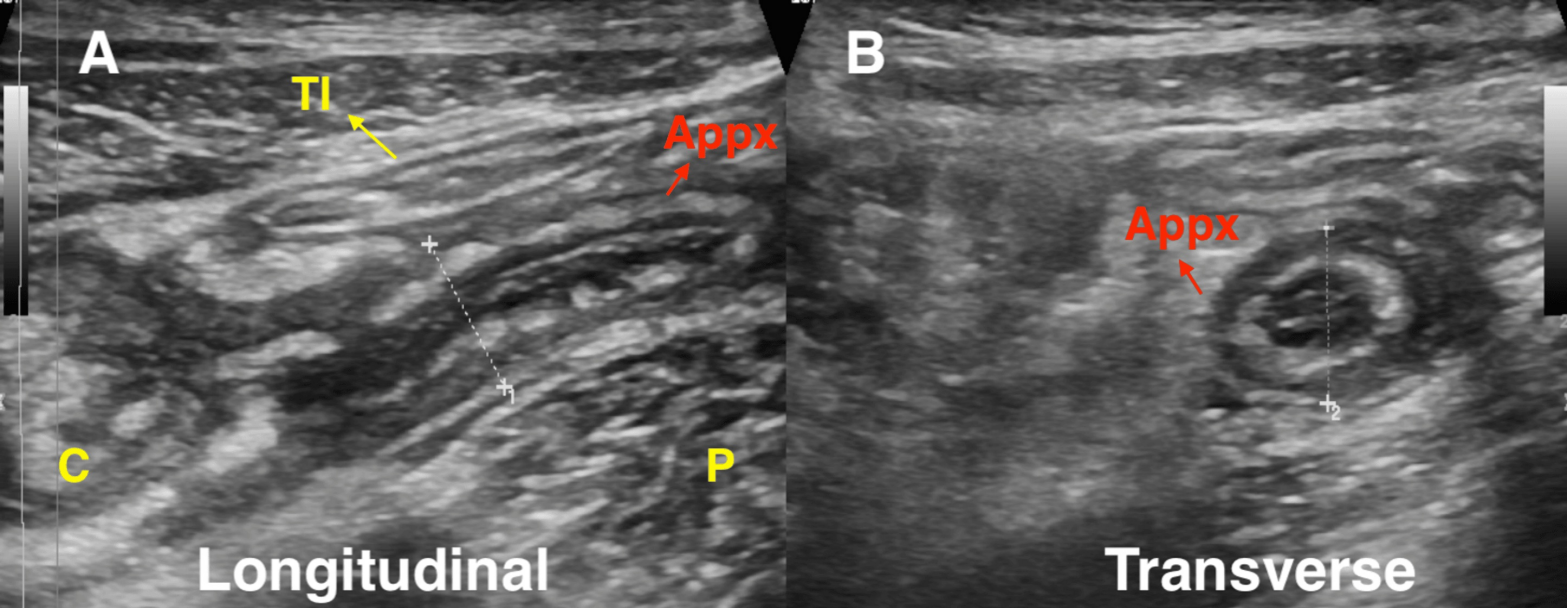
Visualization of the inflamed appendix in USG. (A) The longitudinal axis shows a dilated, tubular, aperistaltic, non-compressible structure in the right iliac fossa arising from the cecum. (B) The transverse axis shows a distended appendix giving a target-like appearance. Original USG image of a patient diagnosed as AA with impending perforation. USG: ultrasound; AA: acute appendicitis; Appx: appendix; C: cecum; P: psoas; TI: terminal ileum

Abdominal USG was performed with a GE-LOGIQ S7 machine and a 3-5 MHz convex transducer. The initial examination aimed to detect abnormalities in solid organs and assess for free fluid in the abdominal cavity. A graded compression technique was utilized for better visualization of the right lower quadrant, concentrating on areas of tenderness. A linear transducer was employed for color Doppler USG to evaluate vascularity. Findings were interpreted by a qualified radiologist, to report if AA is present or not, including various diagnoses such as mesenteric lymphadenitis or free fluid. The appearance of an inflamed appendix is illustrated in Figure 1A, 1B.

CT protocol

CT scans were performed using a 128-slice CT scanner (GE-OPTIMA) with standard settings of 120 kVp and 100 mAs. Patients were administered non-ionic contrast (Iohexol 350) depending on their body weight through an appropriate cannula in the cubital vein.

Sample size calculation

**Figure 2:**
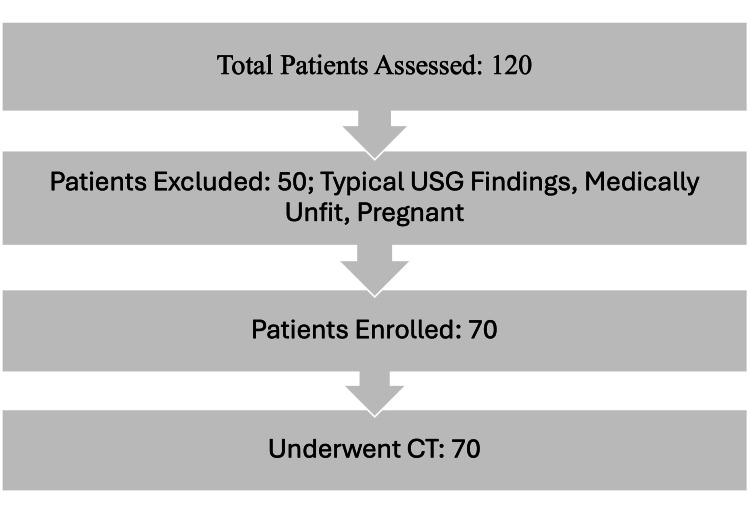
The participant enrolment is illustrated in the flowchart.

The sample size for this study was determined using the formula for estimating proportions in a population. Based on previous literature, the rate of negative findings in USG evaluations for appendicitis was estimated to be approximately 61.5% (p-value) according to Stroman et al. [8], and a sample size of approximately 65 participants was derived. A final sample size of 70 patients was targeted for inclusion in the analysis. The implementation of the methodology for patient selection is illustrated in Figure 2.

Outcomes

The primary outcome was to determine the diagnostic accuracy of CT in identifying appendicitis in patients with negative USG results. This was measured by calculating sensitivity, specificity, PPV, NPV, and overall accuracy. The secondary outcomes included the identification of alternative diagnoses found on CT in patients who were suspected of having appendicitis but were ultimately diagnosed with other conditions, such as mesenteric lymphadenitis, ureteric calculus, and other gastrointestinal or gynecological pathologies.

Statistical analysis

Statistical analysis was performed using SPSS software, and the results were interpreted with a significance level set at (p<0.05). Descriptive statistics such as demographic and clinical characteristics of the study participants were summarized using means, medians, and standard deviations for continuous variables and counts and percentages for categorical variables.

The baseline demographics and clinical characteristics of the study population are summarized in Table 1. 

**Table 1:** Baseline demographics and clinical characteristics of participants.

Characteristics	N (%)
Age group (years)	
0-19	20 (29%)
20-24	6 (8%)
25-80	44 (63%)
Sex	
Male	34 (49%)
Female	36 (51%)
Presenting symptoms	
Abdominal pain	0 (0%)
Fever	70 (100%)
Vomiting	38 (54%)
Constipation	23 (33%)
Loose stool	2 (3%)
Low backache	2 (3%)
Painful micturition	5 (7%)
Ultrasound finding	
Normal	27 (38%)
Mesenteric lymphadenitis	25 (36%)
Free fluid	18 (26%)
Final diagnosis	
Appendicitis	35 (50%)
Other conditions	35 (50%)

Diagnostic performance metrics such as sensitivity, specificity, PPV, NPV, and overall accuracy of CT in diagnosing appendicitis were calculated. Chi-squared tests were utilized to examine the association between categorical variables, such as age, sex, and the presence of appendicitis for inferential statistics. A p-value of <0.05 was accepted as statistically significant in our study.

## Results

The mean age is 35.4 years (SD=15.6). The majority of participants (63%) were aged between 25 and 80 years. The study population comprised almost equal number of males and females, indicating a balanced representation of both sexes. All participants presented with abdominal pain, which was universally reported. Other common symptoms included fever, vomiting, constipation, loose stool, low backache, and painful micturition. Among the 70 patients, 27 had normal USG findings, while 25 exhibited mesenteric lymphadenitis, and 18 showed free fluid in the abdominal cavity. Following CT imaging, 35 patients were diagnosed with appendicitis, while the remaining 35 patients were identified with other conditions, including mesenteric lymphadenitis and ureteric calculus.

The primary and secondary outcomes of the study are summarized below, including the diagnostic performance metrics of CT in identifying appendicitis in patients with negative USG findings.

**Figure 3:**
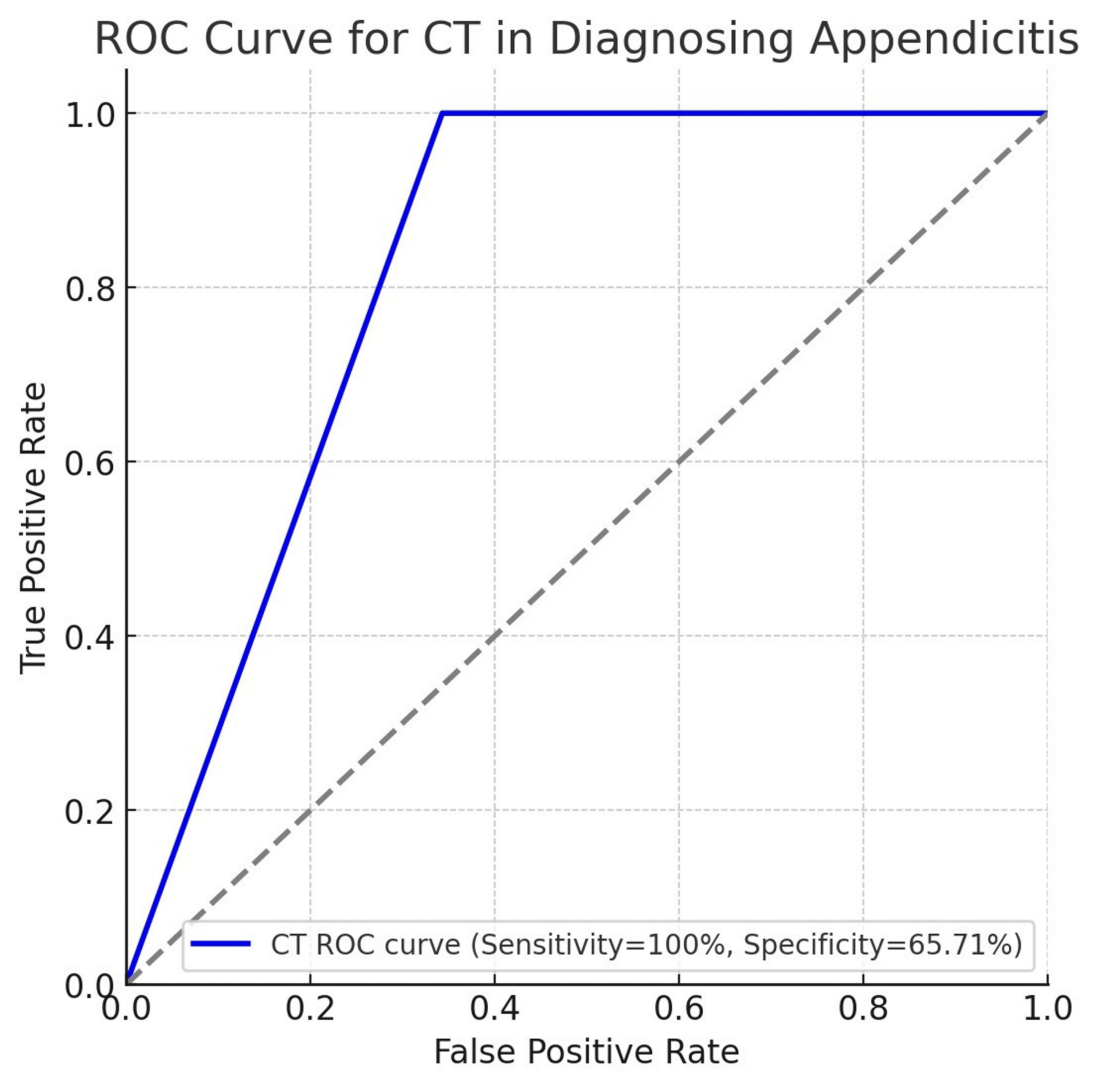
ROC curve for CT in diagnosing appendicitis. CT: computed tomography; ROC: receiver operating characteristic

The overall diagnostic performance of CT in detecting appendicitis is outlined in Table 2. The Receiver operating characteristic (ROC) curve for CT in diagnosing appendicitis is illustrated in Figure 3. The curve shows that CT has excellent sensitivity with a lower specificity, indicating that it correctly identifies all true positives but may also result in some false positives. The analysis revealed a high sensitivity and NPV, indicating that CT is effective in ruling out appendicitis in this cohort.

**Table 2:** Diagnostic performance metrics of CT for appendicitis. CT: computed tomography

Metric	Value
Sensitivity	100%
Specificity	65.71%
Positive predictive value	74.47%
Negative predictive value	100%
Overall accuracy	82.86%

In addition to diagnosing appendicitis, CT imaging identified several alternative diagnoses that contributed to their clinical presentation which are summarized in Table 3. 

**Table 3:** Secondary outcomes: alternative diagnoses identified by CT. CT: computed tomography

Alternative diagnosis (35 cases)	Number of patients N (%)
Mesenteric lymphadenitis	13 (37.14%)
Ureteric calculus	5 (14.29%)
Ileocecal thickening	2 (5.71%)
Oophoritis	1 (2.86%)
Pancreatitis	3 (8.57%)
Normal finding	11 (31.43%)

These results indicate that CT not only plays a critical role in diagnosing appendicitis but also assists in identifying other potential causes of abdominal pain, thus informing clinical decision-making and management strategies.

## Discussion

AA is a prevalent cause of pain in the right lower quadrant of the abdomen. Literature shows that CT scans have a higher accuracy for diagnosing AA as compared to USG [9-11]. "Clinical policy (2010) from the American College of Emergency Physicians on the evaluation and management of emergency department patients with suspected appendicitis" discusses various aspects of CT scans; however, the role of USG in diagnosing appendicitis in adults is not clearly stated [11]. The majority of research articles support the use of CT as a better modality than USG [12]. 

Despite CT having a higher sensitivity to detect urgent conditions in patients having pain abdomen, USG can be considered as a first line of investigation, with subsequent use of CT, only if USG results were equivocal, inconclusive, or negative. This results in excellent sensitivity and also lowers radiation exposure [13]. Even as of today, several reports indicate the use of USG to diagnose AA in the pediatric population [14-17].

In our study, age distribution analysis revealed a significant non-uniformity among the study population, with the chi-squared test yielding a p-value of 0.001. This indicates a statistically significant variation in the ages of patients presenting with right lower quadrant pain. The majority of the patients (63%) fell within the 25-80-year age range, suggesting that this age group is more prone to experiencing right lower quadrant pain and also having equivocal or negative USG findings for appendicitis. This trend might reflect the prevalence of other differential diagnoses in older adults that mimic appendicitis symptoms.

The sex distribution of the study population was nearly equal, comprising 49% male and 51% female patients. The chi-squared test for sex distribution (p=0.811) indicated no significant difference between the sexes regarding the prevalence of right lower quadrant pain. This finding suggests that right lower quadrant pain affects both males and females almost equally, implying that sex is not a significant factor in the occurrence of this symptom.

When comparing the age and sex groups, the analysis found no significant association, with a p-value of 0.639. This indicates that the distribution of right lower quadrant pain across different age groups does not significantly differ between males and females. Therefore, the likelihood of experiencing right lower quadrant pain is similar across all age groups for both sexes.

Abdominal pain was the universal symptom, present in all patients. Other common symptoms included fever and vomiting, while less common symptoms were constipation, loose stool, low backache, and painful micturition. The presence of fever was noted in 54% of patients. The chi-squared test (p=0.398) showed no significant difference in the prevalence of fever among the patients. This finding suggests that fever is not a distinguishing factor among patients presenting with right lower quadrant pain and does not significantly aid in differentiating between appendicitis and other conditions. This spectrum of symptoms indicates that right lower quadrant pain often presents with a variety of associated complaints, which can complicate the clinical diagnosis.

Normal USG findings were observed in 38% of the study population. In contrast, 36% had mesenteric lymphadenitis, and 26% had free fluid. These findings suggest that USG may not always be sufficient for diagnosing appendicitis, as a significant portion of patients with right lower quadrant pain and negative USG results have other underlying conditions.

In patients with negative USG findings, CT scans revealed that 80% of patients had a retrocecal appendix, while smaller percentages had pelvic (7%), pre-/post-ileal (6%), and other positions (7%). The predominance of the retrocecal appendix position among these patients suggests that this anatomical variation might be a common factor in those presenting with right lower quadrant pain.

Pinto Leite et al. proposed that "an appendix diameter of less than 6 mm, or a diameter of 6-10 mm with a gas-filled appendix, or 6-10 mm without other CT signs should be considered as possible appendicitis. A diameter of 6-10 mm with wall thickening (greater than 3 mm) and wall hyper-enhancement, with or without fat stranding, was categorized as probable appendicitis. An appendix diameter greater than 10 mm, or 6-10 mm with wall thickening and hyper-enhancement along with fat stranding, was categorized as definite appendicitis" [18]. Appendix diameter varied among patients, with significant findings indicating that larger diameters are associated with a higher likelihood of appendicitis. Specifically, patients with an appendix diameter ≥6 mm were more likely to have appendicitis, and those with a diameter >8 mm showed an even higher likelihood. This highlights the importance of measuring appendix diameter in the diagnostic process.

A study by Martin et al. showed that CT has a vital role in diagnosing and managing appendicular masses [19]. CT features of inflammatory appendicular mass included walled-off appendicular perforation, phlegmon, and the involvement of adjacent bowel loops such as the cecum and terminal ileum and sometimes other viscera.

The recommended management strategy for complicated cases is the Ochsner method [20], followed by interval appendectomy 6-12 weeks later, which is considered the gold standard. This method is shown to reduce surgical complications.

Choi et al. stated that complications of appendicitis, such as appendiceal perforation, abscess, peritonitis, bowel obstruction, and gangrenous appendicitis, are mostly assessed by USG [21]. However, in some ambiguous cases, CT is essential for diagnosis. Choi et al. also stated that visualization of appendicolith on CT increases the probability of perforation of the appendix as the presence of an appendicolith raises the risk of perforation [21].

**Figure 4:**
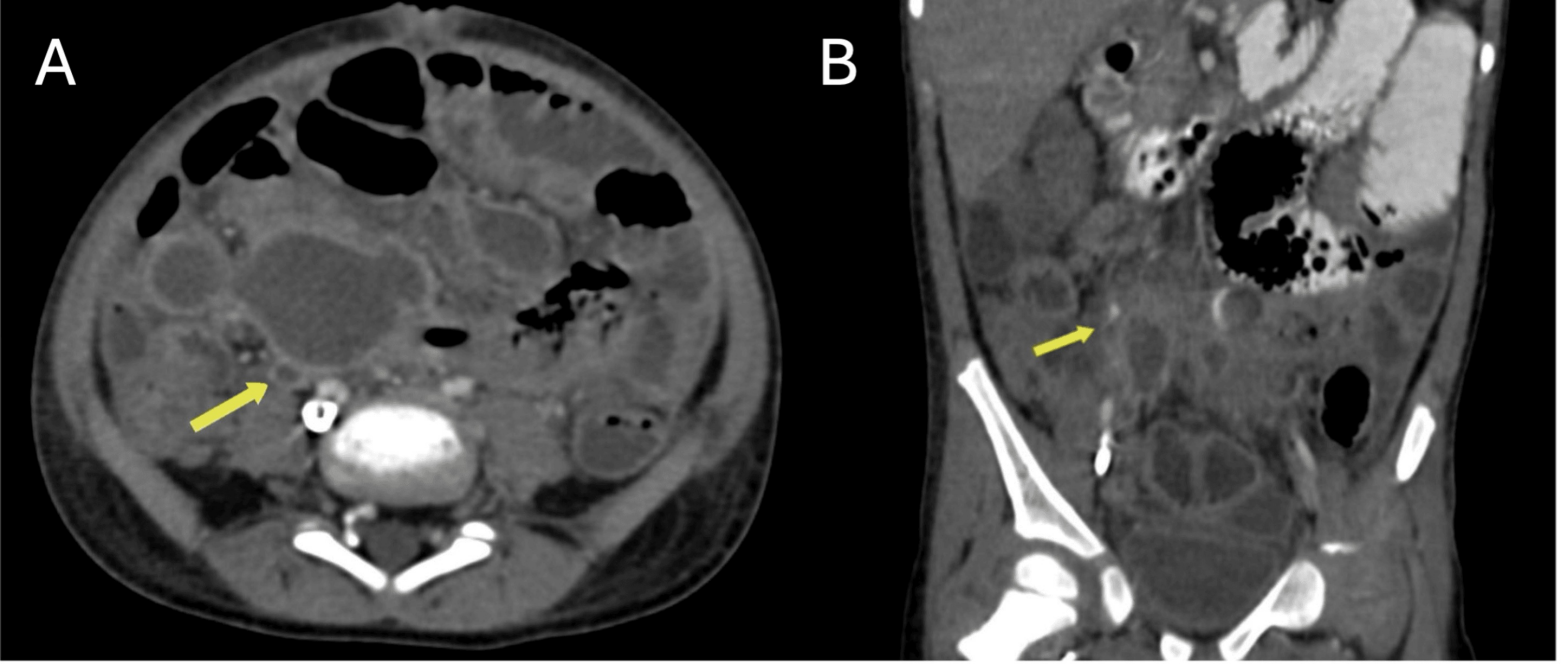
CT of a 13-year-old male with right iliac fossa pain. (A) Axial CT shows a hyper-dense appendicolith. (B) Coronal CT shows a dilated tubular structure in the right iliac fossa with surrounding inflammatory changes, consistent with appendicitis. CT: computed tomography

In our study, appendicular perforation or abscess was present in 19% of patients, highlighting a significant complication in a subset of the study population. Therefore, the presence of one or more appendicoliths with periappendiceal inflammation is almost diagnostic of perforation or impending perforation as can be seen in Figure 4A, 4B.

Differential diagnoses in patients with right lower quadrant pain included mesenteric adenitis, epiploic appendagitis, cecal diverticulitis, Crohn's disease, omental infarction, perforated cecal carcinoma, infectious terminal ileitis, appendiceal mucocele, and appendiceal carcinoma. Mesenteric lymphadenitis is characterized by enlarged lymph nodes in the mesentery, often associated with gastrointestinal infections. It was commonly misdiagnosed as appendicitis based on clinical symptoms. Ureteric calculus caused significant pain that mimicked appendicitis, thus highlighting the importance of differential diagnosis in patients presenting with right lower quadrant pain. Ileocecal thickening can indicate inflammatory bowel disease or other gastrointestinal pathology, which may present similarly to appendicitis. Oophoritis indicates inflammation of the ovaries, which can occur in females presenting with right-sided abdominal pain. Pancreatitis may present with abdominal pain and elevated inflammatory markers, further complicating the diagnostic process.

These additional findings underscore the complexity of diagnosing appendicitis and highlight the importance of comprehensive imaging evaluation in patients with ambiguous clinical presentations. The ability of CT to reveal alternative diagnoses provides critical information that aids in appropriate patient management.

Coursey et al. [22] proposed that "although CT carries a risk of radiation exposure, the estimated lifetime attributable risk of death from cancer is between 0.05% and 0.06% for a 25-year-old patient undergoing abdominal CT with a current setting of 240 mAs." However, the average mortality for a negative appendectomy is 0.14%, for AA is 0.24%, and for perforated appendicitis is 1.66%. Therefore, we believe the risk of unnecessary surgical interventions and mortality justifies the use of CT in equivocal or uncertain cases. Wagner et al. found that preoperative CT imaging reduces the rate of negative appendectomy from 16% to 5% in both adults and children without increasing the rate of perforation [23].

The management of right iliac fossa pain varied among the study population in our study, with 43% of patients undergoing surgery and 57% receiving conservative treatment. For patients specifically diagnosed with appendicitis, 86% were treated surgically, indicating a significant reliance on surgical intervention for confirmed cases (p=0.001). All patients with appendicular perforation or abscess underwent surgery, emphasizing the need for prompt surgical management in complicated cases.

The study concludes that CT is highly effective in diagnosing appendicitis in patients with right lower quadrant pain and negative USG findings. The statistical analysis highlights that larger appendix diameters (≥6 mm) are significantly associated with appendicitis, making CT an invaluable tool in the diagnostic process. The management approach is predominantly surgical for confirmed appendicitis, while conservative treatment is more common for other conditions presenting with similar symptoms. The findings underscore the need for accurate imaging and prompt intervention to ensure appropriate management and improve patient outcomes.

Study limitations and future directions

Despite the strong findings of this study, there remain limitations that warrant consideration. The study population was relatively small, and it was conducted at a single institution, which may limit the generalizability of the results. Future research should explore larger, multi-center studies to further validate these results, assess long-term outcomes associated with CT use in appendicitis diagnosis, and investigate patient factors that may influence the relationship between imaging findings and clinical outcomes

## Conclusions

The findings of this study affirm the crucial role of CT in diagnosing appendicitis in USG-negative patients. By enhancing diagnostic accuracy and reducing unnecessary surgeries, CT reinforces its position as a cornerstone of modern diagnostic imaging in appendicitis cases. Embracing an integrated approach that combines conventional imaging with advanced modalities such as CT can significantly improve clinical outcomes, ultimately leading to better patient care in emergency settings. Future efforts in imaging research and practice should prioritize optimizing diagnostic strategies for AA, with an aim towards minimizing risks while maximizing diagnostic efficacy.
